# Supplementary material for: Taking a shortcut: what mechanisms do fish use?
Source: Commun Biol. 2024 May 16;7:578. doi: 10.1038/s42003-024-06179-5 (PMC11099040; doi:10.1038/s42003-024-06179-5)
Supplement: Supplementary file 2 — Supplementary information [file 42003_2024_6179_MOESM2_ESM.pdf]

Supplementary information_ Taking a shortcut: what mechanisms do fish use? (Sibeaux, Newport, Green, Karlsson, Engelmann & Burt de Perera)

Supplementary Table S1: Experimental setup. Cichlids Group 1 experienced displacement Lateral first and a Diagonal displacement, two weeks after. Group 2 experienced a Diagonal displacement first and a Lateral displacement two weeks after.

Weekday	Day	Fish	Group	Displacement	Day	Fish	Group	Displacement	Day	Fish	Group	Displacement	Day	Fish	Group	Displacement
Sunday	1				30				59				86			
Monday	2	C6			31	C11			60	C21			87	C31*		
Tuesday	3	C7			32	C12			61	C22			88	C32		
Wednesday	4	C8	1	Lateral	33	C13	1	Lateral	62	C23	1	Lateral	89	C33	1	Lateral
Thursday	5	C9			34	C14			63	C24			90	C34		
Friday	6	C10			35	C15			64	C25			91	C35*		
Saturday	7				36				65				92			
Sunday	8				37				66				93			
Monday	9	C1			38	C16			67	C26			94	C36		
Tuesday	10	C2			39	C17			68	C27			95	C37		
Wednesday	11	C3	2	Diagonal	40	C18	2	Diagonal	69	C28	2	Diagonal	96	C38	2	Diagonal
Thursday	12	C4			41	C19			70	C29			97	C39d**		
Friday	13	C5			42	C20			71	C30			98	C40		
Saturday	14				43				72				99			
Sunday	15				44				73				100			
Monday	16	C6			45	C11			74	C21			101	C31	2	
Tuesday	17	C7			46	C12			75	C22			102	C32		
Wednesday	18	C8	1	Diagonal	47	C13	1	Diagonal	76	C23	1	Diagonal	103	C33	1	Diagonal
Thursday	19	C9			48	C14			77	C24			104	C34		
Friday	20	C10			49	C15			78	C25			105	C35	2	
Saturday	21				50				79				106			
Sunday	22				51				80				107			
Monday	23	C1			52	C16			81	C26			108	C36		
Tuesday	24	C2			53	C17			82	C27			109	C37	2	
Wednesday	25	C3	2	Lateral	54	C18	2	Lateral	83	C28	2	Lateral	110	C38		Lateral
Thursday	26	C4			55	C19			84	C29			111	C39	1	
Friday	27	C5			56	C20			85	C30			112	C40	2	
													113			
													114			
													115	C31	2	Lateral
													116	C35	2	
													117	C39	1	Diagonal

* Building work in the lab next door could have alter the fish behaviour; ** individual C39d died between the two sessions

Supplementary Tables S2: Results from the the Circ_MLE analysis.

Supplementary Table S2a: **Fish First orientation circular analysis.** Output from CircMLE v0.2.0. The output from the 'circ_mle' function for all 10 models of orientation using default parameters. The input data consisted of the 37 orientation trajectories when the fish just exited the reward chamber. We associated the circular figure for our dataset below the table (grey bars= data histogram, red arrow= mean angle). The density (dotted line) and mean angles (dotted arrow) for the best-fit model M5A are shown.

M1 = Uniform, M2A = Unimodal, M2B = Symmetric modified unimodal, M2C = Modified unimodal, M3A = Homogenous symmetric Bimodal, M3B = Symmetric bimodal, M4A = Homogenous axial bimodal, M4B = Axial bimodal 4, M5A = Homogenous bimodal, M5B = Bimodal. For model definition see table 1 in (Fitak & Johnsen, 2017).

	M5A	M2A	M2C	M2B	M3B	M4B	M5B	M4A	M3A	M1
Params[1]	4	2	3	2	3	4	5	3	2	0
**$\varphi 1$**[2]	0.61	1.87	2.11	2.13	5.26	5.43	6.28	5.11	2.27	NA
**$\kappa 1$**[3]	4.63	1.98	3.77	4.18	0.00	0.00	5.95	2.00	1.82	0.00
λ	0.25	1.00	0.75	0.50	0.50	0.35	0.40	0.25	0.50	1.00
**$\varphi 2$**	2.19	NA	NA	NA	8.41	8.57	2.23	8.25	5.41	NA
**$\kappa 2$**	4.63	0.00	0.00	0.00	4.54	5.00	5.06	2.00	1.82	0.00
Likelihood[4]	42.63	47.13	50.24	52.32	52.35	52.06	51.18	55.08	65.21	68.00
Convergence[5]	0	0	0	0	0	0	0	0	0	0
AIC	93.26	98.26	106.48	108.64	110.70	112.12	112.36	116.16	134.41	136.00
AICc	94.51	98.61	107.21	109.00	111.42	113.37	114.30	116.88	134.77	136.00
BIC	99.70	101.48	111.31	111.87	115.53	118.57	120.42	120.99	137.64	136.00
ΔAIC	0.00	5.00	13.22	15.39	17.44	18.87	19.11	22.90	41.16	42.75
ΔAICc	0.00	4.10	12.70	14.49	16.92	18.87	19.79	22.38	40.26	41.50
ΔBIC	0.00	1.78	11.61	12.17	15.83	18.87	20.72	21.29	37.94	36.30
Relative likelihoods	1.00	0.08	0.00	0.00	0.00	0.00	0.00	0.00	0.00	0.00
AIC weights	0.92	0.08	0.00	0.00	0.00	0.00	0.00	0.00	0.00	0.00
Evidence Ratio	NA	1.22E+01	7.43E+02	2.19E+03	6.12E+03	1.25E+04	1.41E+04	9.38E+04	8.65E+08	1.92E+09

[1] Params = number of free parameters in the model.

[2] φi = Mean direction (in radians)

[3] κi = Concentration parameters

[4] Negative log likelihood

[5] Whether or not the maximum likelihood search converged, 0= convergence

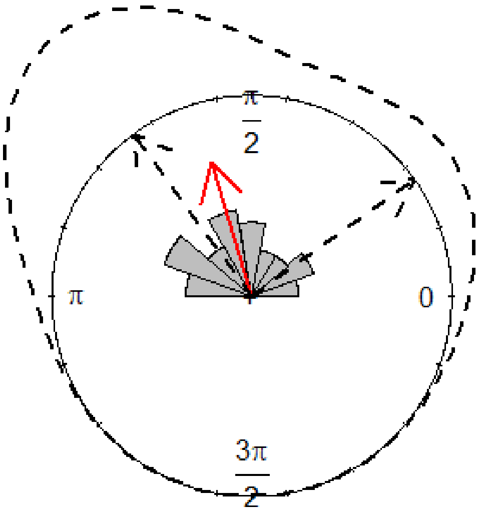

Figure associated with table S2a: Raw plot from the circ_mle function. The original parameters from the plot have been left intact meaning that the angular values are in radians units and the values are plotted counter clockwise.

Supplementary Table S2b: **Angle start-end circular analysis.** Output from CircMLE v0.2.0. The output from the 'circ_mle' function for all 10 models of orientation using default parameters. The input data consisted of the 37 orientation trajectories of the fish full trajectory (angle start-end). We associated the circular figure for our dataset below the table (grey bars= data histogram, red arrow= mean angle). The density (dotted line) and mean angles (dotted arrow) for the best-fit model M5B are shown.

M1 = Uniform, M2A = Unimodal, M2B = Symmetric modified unimodal, M2C = Modified unimodal, M3A = Homogenous symmetric Bimodal, M3B = Symmetric bimodal, M4A = Homogenous axial bimodal, M4B = Axial bimodal 4, M5A = Homogenous bimodal, M5B = Bimodal. For model definition see table 1 in (Fitak & Johnsen, 2017).

	M5B	M5A	M2A	M2C	M2B	M3B	M4B	M4A	M1	M3A
Params[1]	5	4	2	3	2	3	4	3	0	2
**φ1**[2]	2.65	2.58	1.69	1.54	1.79	5.35	1.64	2.65	NA	0.00
**κ1**[3]	11.66	5.89	1.35	1.89	1.61	0.00	1.94	1.38	0.00	1.54
λ	0.46	0.50	1.00	0.75	0.50	0.50	0.75	0.75	1.00	0.50
**φ2**	0.83	0.75	NA	NA	NA	8.49	4.78	5.79	NA	3.14
**κ2**	4.00	5.89	0.00	0.00	0.00	2.41	0.96	1.38	0.00	1.54
Likelihood[4]	43.58	44.85	55.49	58.66	60.30	61.30	62.19	64.77	68.00	66.34
Convergence[5]	0	0	0	0	0	0	0	0	0	0
AIC	97.17	97.69	114.97	123.31	124.61	128.59	132.38	135.55	136.00	136.68
AICc	99.10	98.94	115.33	124.04	124.96	129.32	133.63	136.27	136.00	137.03
BIC	105.22	104.13	118.19	128.15	127.83	133.43	138.82	140.38	136.00	139.90
ΔAIC	0.00	0.53	17.81	26.15	27.44	31.43	35.21	38.38	38.84	39.51
ΔAICc	0.16	0.00	16.39	25.10	26.02	30.38	34.69	37.33	37.06	38.09
ΔBIC	1.09	0.00	14.06	24.01	23.70	29.29	34.69	36.24	31.87	35.77
Relative likelihoods	1.00	0.77	0.00	0.00	0.00	0.00	0.00	0.00	0.00	0.00
AIC weights	0.57	0.43	0.00	0.00	0.00	0.00	0.00	0.00	0.00	0.00
Evidence Ratio	NA	1.30E+00	7.36E+03	4.77E+05	9.10E+05	6.68E+06	4.42E+07	2.16E+08	2.71E+08	3.80E+08

[1] Params = number of free parameters in the model.

[2] **φi** = Mean direction (in radians)

[3] **κi** = Concentration parameters

[4] Negative log likelihood

[5] Whether or not the maximum likelihood search converged, 0= convergence

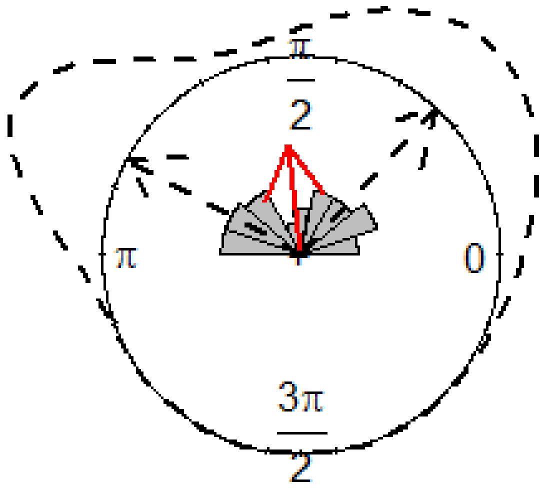

Figure associated with table S2b: Raw plot from the circ_mle function. The original parameters from the plot have been left intact meaning that the angular values are in radians units and the values are plotted counter clockwise.

Supplementary Table S3: Average distance between the fish trajectory and the model trajectories and between fish trajectories and the randomly generated model trajectories. The smallest average distance between the fish trajectory and one of the three model trajectories is in bold.

	Fish	Distance to PI (mean±SD)	Distance to APC (mean±SD)	Distance to RR (mean±SD)	AIC	BIC	Average ± $SD_{uniform}$ 10000 Random
**Diagonal displacement**	1D	58.93 ± 35.40^{a}	92.22 ± 45.10^{b}	**50.25 ± 55.31**^{c}	31491.52	31521.55	61.14 ± 26.19
	3D	**47.02 ± 36.12**^{a}	89.33 ± 40.14^{b}	52.45 ± 30.70^{c}	29998.6	30028.63	61.89 ± 22.58
	4D	67.47 ± 45.94^{a}	82.01 ± 39.98^{b}	**62.12 ± 55.98**^{c}	31717.7	31747.74	59.44 ± 27.99
	6D	73.81 ± 43.12^{a}	69.98 ± 41.43^{b}	**52.43 ± 41.52**^{c}	30951.05	30981.08	60.68 ± 33.81
	10D	122.30 ± 73.69^{a}	**21.51 ± 12.28**^{b}	124.74 ± 82.66^{a}	33504.32	33534.36	79.92 ± 37.00
	12D	98.82 ± 49.91^{a}	**46.49 ± 33.39**^{b}	70.94 ± 36.51^{c}	30739.84	30769.87	65.29 ± 35.67
	14D	44.24 ± 24.74^{a}	75.10 ± 40.07^{b}	**12.03 ± 7.84**^{c}	28418.93	28448.96	41.70 ± 19.25
	18D	43.44 ± 22.59^{a}	33.07 ± 10.30^{b}	**22.08 ± 12.48**^{c}	25172.44	25202.47	30.17 ± 14.34
	20D	31.61 ± 16.19^{a}	64.08 ± 35.98^{b}	**10.57 ± 8.16**^{c}	27400.98	27431.01	34.77 ± 15.72
	21D	**27.37 ± 13.60**^{a}	111.53 ± 51.88^{b}	65.41 ± 18.88^{c}	29468.34	29498.37	67.17 ± 35.74
	22D	**13.64 ± 11.17**^{a}	94.68 ± 48.88^{b}	32.42 ± 11.22^{c}	28860.8	28890.83	45.40 ± 24.35
	25D	18.99 ± 11.45^{a}	59.82 ± 36.75^{b}	**14.15 ± 5.13**^{c}	27180.84	27210.87	30.06 ± 15.22
	26D	52.64 ± 30.21^{a}	**8.22 ± 3.30**^{b}	37.91 ± 22.61^{c}	27031.52	27061.55	35.22 ± 15.52
	29D	94.53 ± 64.50^{a}	**58.40 ± 31.70**^{b}	86.35 ± 69.25^{c}	32844.24	32874.27	64.69 ± 35.53
	31D	**29.23 ± 10.72**^{a}	87.10 ± 58.09^{b}	49.71 ± 27.99^{c}	30304.99	30335.03	62.21 ± 33.24
	33D	110.14 ± 50.00^{a}	**61.92 ± 37.75**^{b}	88.53 ± 60.21^{c}	32013.06	32043.1	76.58 ± 38.99
	35D	**44.00 ± 27.90**^{a}	84.52 ± 50.39^{b}	49.40 ± 39.43^{c}	30699.01	30729.04	59.62 ± 27.49
	39D	77.05 ± 45.15^{a}	79.82 ± 44.72^{a}	**56.76 ± 56.97**^{b}	31905.09	31935.12	61.73 ± 31.94
	**Mean Diagonal**	**58.62 ± 49.44**	**67.77 ± 47.31**	**52.13 ± 51.11**			**55.43 ± 42.73**
**Lateral displacement**	1L	80.58 ± 38.37^{a}	**45.42 ± 22.23**	57.88 ± 45.67^{c}	30145.97	30176.01	63.74 ± 32.61
	3L	166.55 ± 79.77^{a}	**64.09 ± 26.41**	161.17 ± 66.68^{a}	33276.98	33307.01	114.8 ± 45.90
	4L	84.39 ± 56.42^{a}	**38.85 ± 11.99**^{b}	85.50 ± 54.05^{a}	31445.03	31475.07	60.94 ± 31.86
	5L	**12.95 ± 5.36**^{a}	43.80 ± 17.90	21.36 ± 7.08^{c}	23192.87	23222.9	30.68 ± 14.87
	6L	48.54 ± 16.25^{a}	68.84 ± 54.71	**31.15 ± 15.82**^{c}	29713.02	29743.05	61.16 ± 22.18
	15L	60.36 ± 34.85^{a}	63.04 ± 36.37	**46.90 ± 50.53**^{b}	30830.14	30860.17	60.26 ± 32.87
	16L	**19.93 ± 10.66**^{a}	57.99 ± 15.15	39.06 ± 7.06^{c}	23147.27	23177.3	41.52 ± 15.00
	18L	**25.86 ± 14.50**^{a}	97.03 ± 54.38	41.53 ± 22.72^{c}	29859.72	29889.76	67.10 ± 34.66
	19L	**18.98 ± 11.13**^{a}	100.10 ± 66.30	51.08 ± 19.32^{c}	30710.95	30740.98	67.88 ± 32.33
	21L	**36.98 ± 21.38**^{a}	93.43 ± 54.03	70.71 ± 40.91^{c}	30806	30836.03	63.51 ± 29.34
	22L	73.06 ± 52.54^{a}	49.51 ± 24.93	**52.84 ± 29.92**^{c}	30308.29	30338.33	63.79 ± 18.59
	26L	**55.06 ± 31.61**^{a}	68.60 ± 34.33	68.83 ± 41.29^{b}	30018.02	30048.05	63.08 ± 16.66
	27L	**42.34 ± 22.52**^{a}	78.78 ± 42.36	60.69 ± 43.31^{c}	30237.23	30267.26	62.66 ± 23.33
	29L	110.17 ± 81.24^{a}	**58.10 ± 31.31**	174.36 ± 102.01^{c}	34616.94	34646.97	83.72 ± 23.48
	31L	106.11 ± 62.18^{a}	**14.45 ± 6.15**^{b}	103.61 ± 44.04^{a}	31244.15	31274.18	69.22 ± 39.03
	33L	90.17 ± 58.25^{a}	**24.84 ± 11.37**	85.12 ± 68.00^{c}	32240.73	32270.76	62.92 ± 31.86
	34L	45.05 ± 10.41^{a}	83.11 ± 25.39	**36.47 ± 9.66**^{c}	25446.95	25476.98	66.25 ± 27.12
	35L	**30.32 ± 10.18**^{a}	71.80 ± 31.58	38.15 ± 17.71^{c}	26987.52	27017.55	54.10 ± 22.12
	39L	**8.60 ± 6.03**^{a}	46.55 ± 30.24	24.30 ± 15.16^{c}	26445.61	26475.64	31.41 ± 16.19
	**Mean Lateral**	**57.31 ± 56.46**	**62.75 ± 42.72**	**64.73 ± 60.28**			**62.57 ± 53.77**

PI= Path Integration, APC=Allothetic place cues, RR= Route recapitulation. For each fish, different letters indicate that the distance between the fish trajectory and the models (PI, APC and RR) were significant different. Each line shows the result of a single linear mixed model (package glmmTMB) with distance as the response variable and model as the explanatory variable. Model was also added as a random intercept. We used the packages "multcomp" to adjust p-values (Holm-Bonferroni correction) and "DHARMa" for model validation. AIC and BIC values are provided for each separate model. The distance to the 10000 randomly generated models followed a uniform distribution. Therefore, the standard deviation for uniform distribution was calculated as $SD_{uniform}$ =(b-a)/sqrt (12), where [a,b] is the interval over which the continuous uniform distribution is defined.

Supplementary Table S4: Fish subpopulations. a) Total number of trials tested fish from each population (each fish was tested twice, therefore Trial tests is the number of fish in each population x 2) and the number of fish that actually entered and exited the reward chamber giving us data to analyse. B) The number of fish from each population that followed either Path integration (PI), Allothetic place cues (APC), Route recapitulation (RR) or showed random movements.

	Population					
	**b**	**c**	**d**	**e**	**f**	**g**
a) **Total**						
Trial tests	8	14	2	8	32	16
Gave us data	5	5	2	2	16	7
b) **Following strategy**						
PI	1	1			4	2
APC			1	1	3	1
RR	1	1			2	1
Random	3	3	1	1	7	3

Testing the effect of subpopulation on individual's chosen strategy was not possible with the multinomial mixed effects regression ("mblogit" function, mclogit package; Elff, 2022), detailed in the methods paragraph "*Testing the effect of age, speed, trial number and displacement on chosen strategy*" due to convergence problems arising from the unequal numbers of individuals sampled from each of the subpopulations.

Supplementary Table S5: Average ± SD path length and straightness for the fish grouped under Path integration (PI), Allothetic place cues (APC), Route recapitulation (RR) and Random trajectories groups.

	PI	APC-Diagonal	APC-lateral	RR	Random
path length	41.81 ± 41.38	145.91 ± 185.63	94.24 ± 75.04	25.59 ± 8.65	79.14 ± 56.42
path straightness	0.89 ± 0.08	0.70 ± 0.38	0.72 ± 0.28	0.92 ± 0.06	0.85 ± 0.10

Supplementary Table S6: Effect of fish age, swimming speed, the test trial number or the displacement on the navigational strategy chosen. The results in bold indicate the significant p-values.

contrast	Fixed effects		β coefficient	SE	z ratio	P
PI-APC	Age		-0.10	0.13	-0.78	1.000
PI-RR			-0.03	0.12	-0.25	1.000
**PI-Random**			-0.47	0.15	-3.11	**0.011**
APC-RR			0.07	0.13	0.55	1.000
APC-Random			-0.37	0.16	-2.34	0.077
**RR-Random**			-0.44	0.17	-2.59	**0.048**
PI-APC	Swimming Speed		-0.10	0.13	-0.78	1.000
PI-RR			-0.03	0.12	-0.25	1.000
**PI-Random**			-0.47	0.15	-3.11	**0.011**
APC-RR			0.07	0.13	0.55	1.000
APC-Random			-0.37	0.16	-2.34	0.077
**RR-Random**			-0.44	0.17	-2.59	**0.048**
PI-APC	Displacement	Diagonal	-0.08	0.09	-0.91	1.000
PI-RR			-0.11	0.11	-0.92	1.000
**PI-Random**			-0.68	0.15	-4.55	**<0.001**
APC-RR			-0.02	0.14	-0.14	1.000
**APC-Random**			-0.60	0.19	-3.16	**0.008**
**RR-Random**			-0.57	0.23	-2.55	**0.043**
PI-APC		Lateral	-0.11	0.21	-0.53	1.000
PI-RR			0.05	0.17	0.29	1.000
PI-Random			-0.26	0.23	-1.16	1.000
APC-RR			0.16	0.18	0.88	1.000
APC-Random			-0.15	0.24	-0.62	1.000
RR-Random			-0.31	0.21	-1.48	0.826
PI-APC	Trial	First	-0.16	0.12	-1.36	0.521
PI-RR			-0.15	0.14	-1.08	0.563
**PI-Random**			-0.63	0.17	-3.76	**0.001**
APC-RR			0.02	0.20	0.08	0.934
APC-Random			-0.46	0.24	-1.89	0.297
RR-Random			-0.48	0.27	-1.75	0.322
PI-APC		Later	-0.03	0.20	-0.16	1.000
PI-RR			0.09	0.16	0.57	1.000
PI-Random			-0.32	0.23	-1.39	0.674
APC-RR			0.12	0.14	0.91	1.000
APC-Random			-0.28	0.19	-1.50	0.674
RR-Random			-0.41	0.17	-2.45	0.085

Pairwise contrast on multinomial regression probabilities with fish identity included as random effect. The results for age and speed were averaged over the levels of displacement and test trial number, the results for displacement was averaged over the levels of test trial number and the results of test trial number was averaged over the levels of displacement. All p-values were adjusted with the Holm-Bonferroni method. β is the coefficient which estimates the strength of the effect, SE is the standard error and Z is an effect size. Random effect= fish ID: n=24. Model null deviance variance=102.6, residual deviance= 73.8.The current version of mblogit package does not allow to obtain contrasts for the intercept and the random effect but information on the full model can be found on the R code (Dryad data).

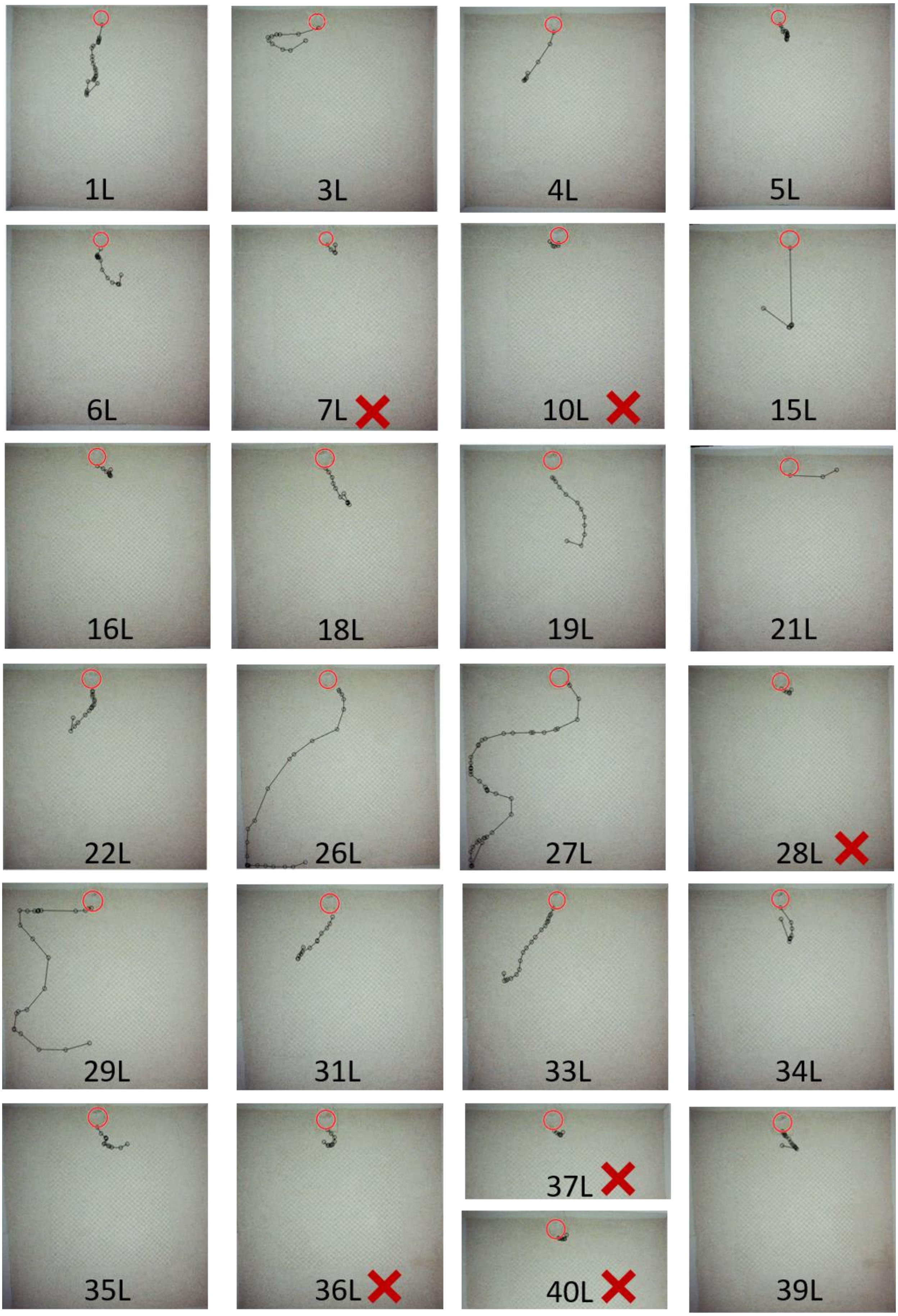

Supplementary Figure S1a: Individual trajectories after Lateral displacement. The red circles indicate the chamber position after displacement. The fish position was collected every second (black dots) and we stopped analysing trajectories when the fish direction oriented toward the origin (chamber). This last position (toward origin) is represented in the following pictures but was not taken into account for the trajectory analysis. The red cross indicates the trials that were discarded (Distance from origin is shorter than the chamber diameter).

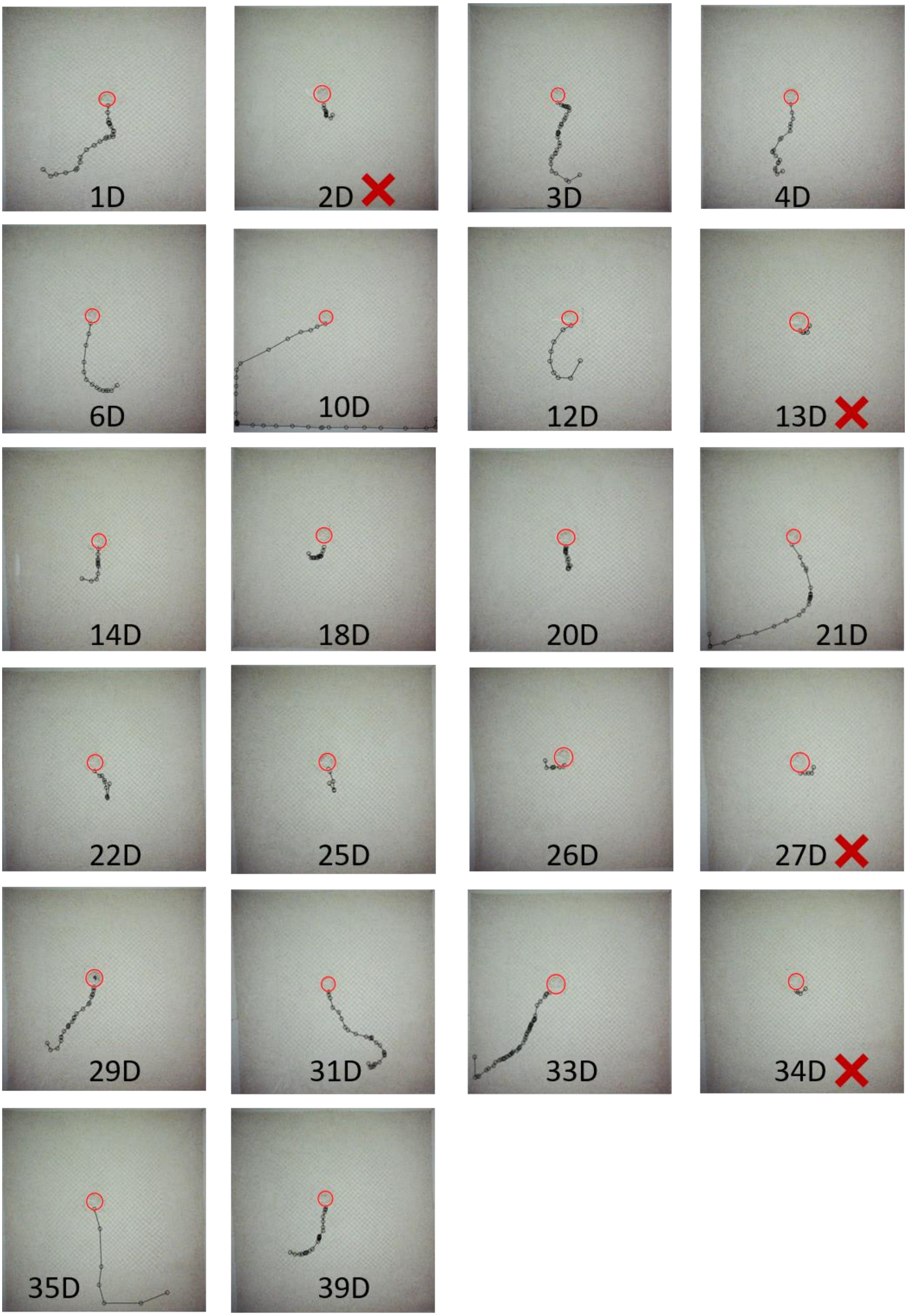

Supplementary Figure S1b: Individual trajectories after Lateral displacement. The red circles indicate the chamber position after displacement. The fish position was collected every second (black circle) and we stopped analysing trajectories when the fish direction oriented toward the origin (chamber). This last position is represented in the following pictures but was not taken into account for the trajectory analysis. The red cross indicates the trials that were discarded (Distance from origin is shorter than the chamber diameter).

1L

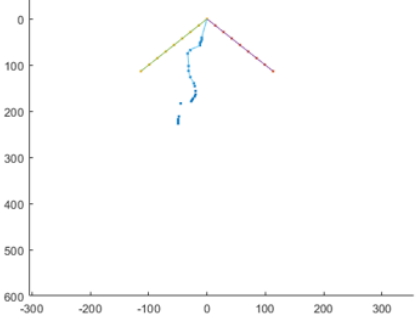

1L

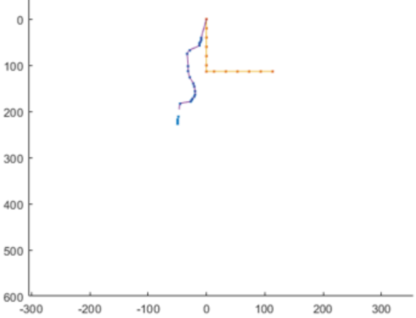

3L

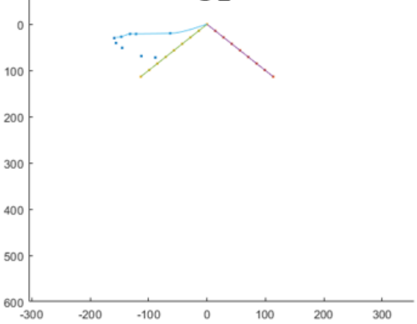

3L

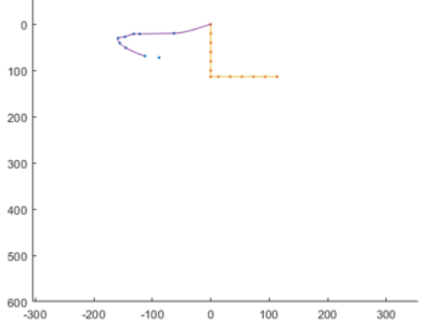

4L

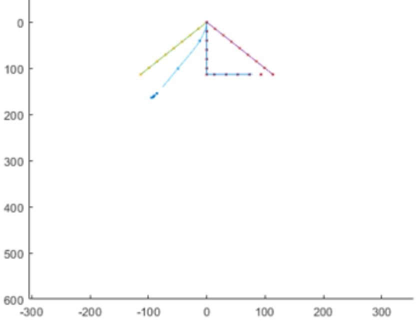

5L

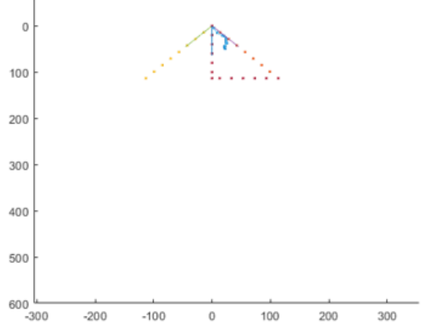

6L

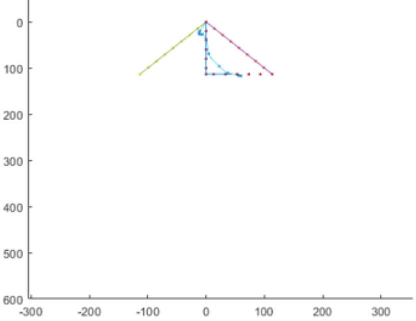

15L

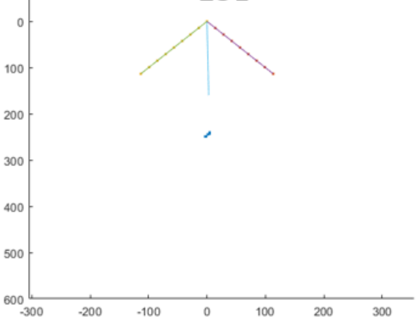

15L

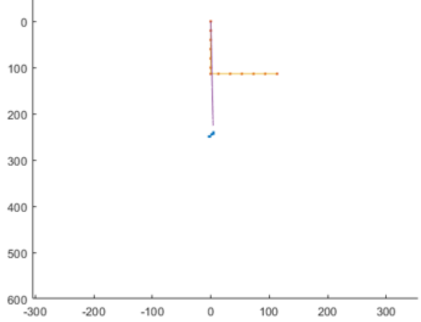

16L

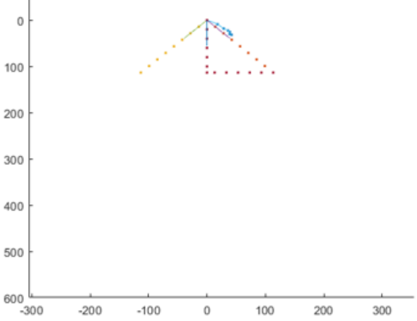

18L

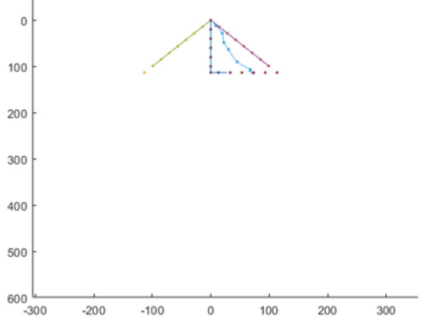

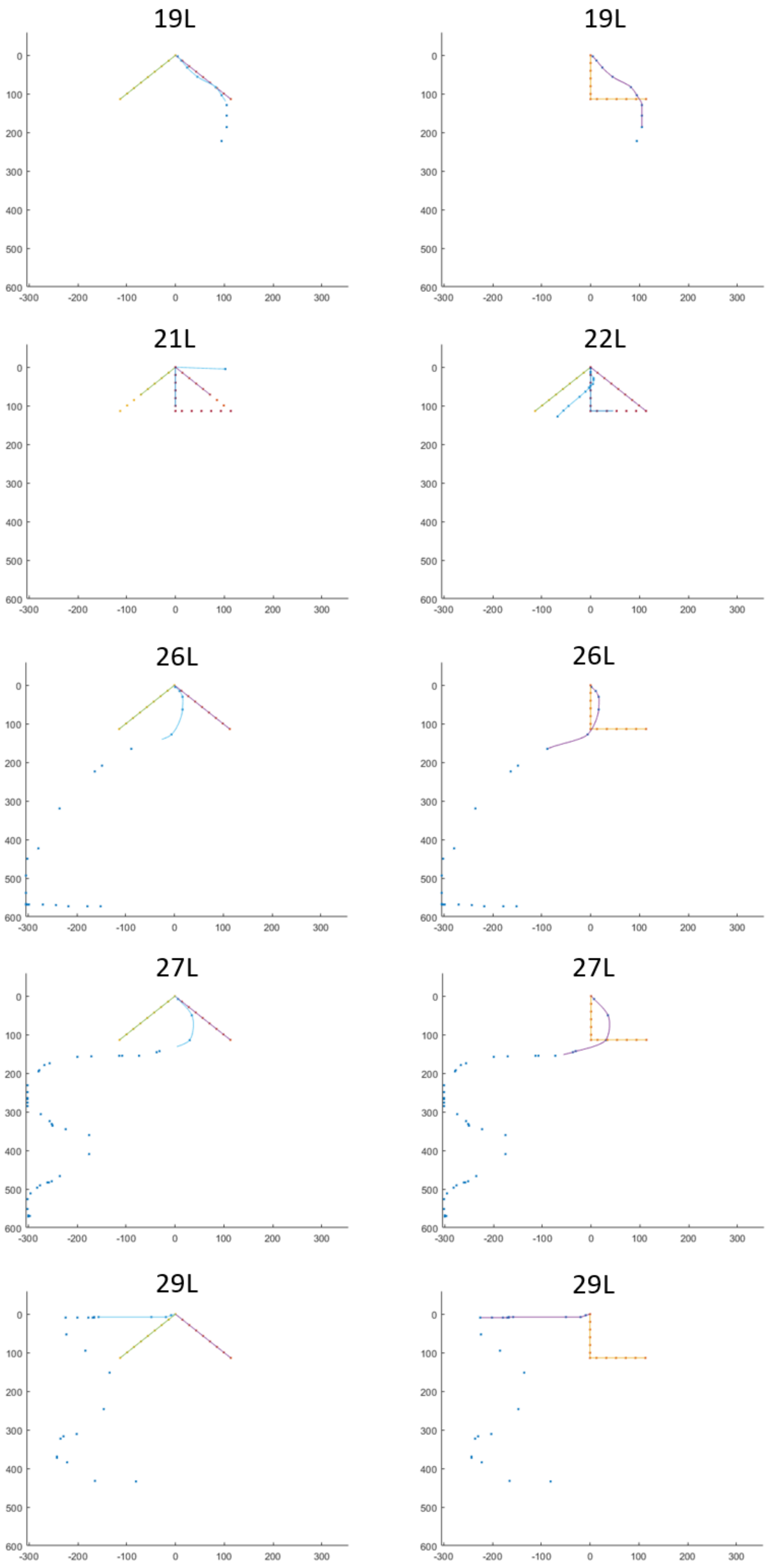
19L
19L
21L
22L
26L
26L
27L
27L
29L
29L

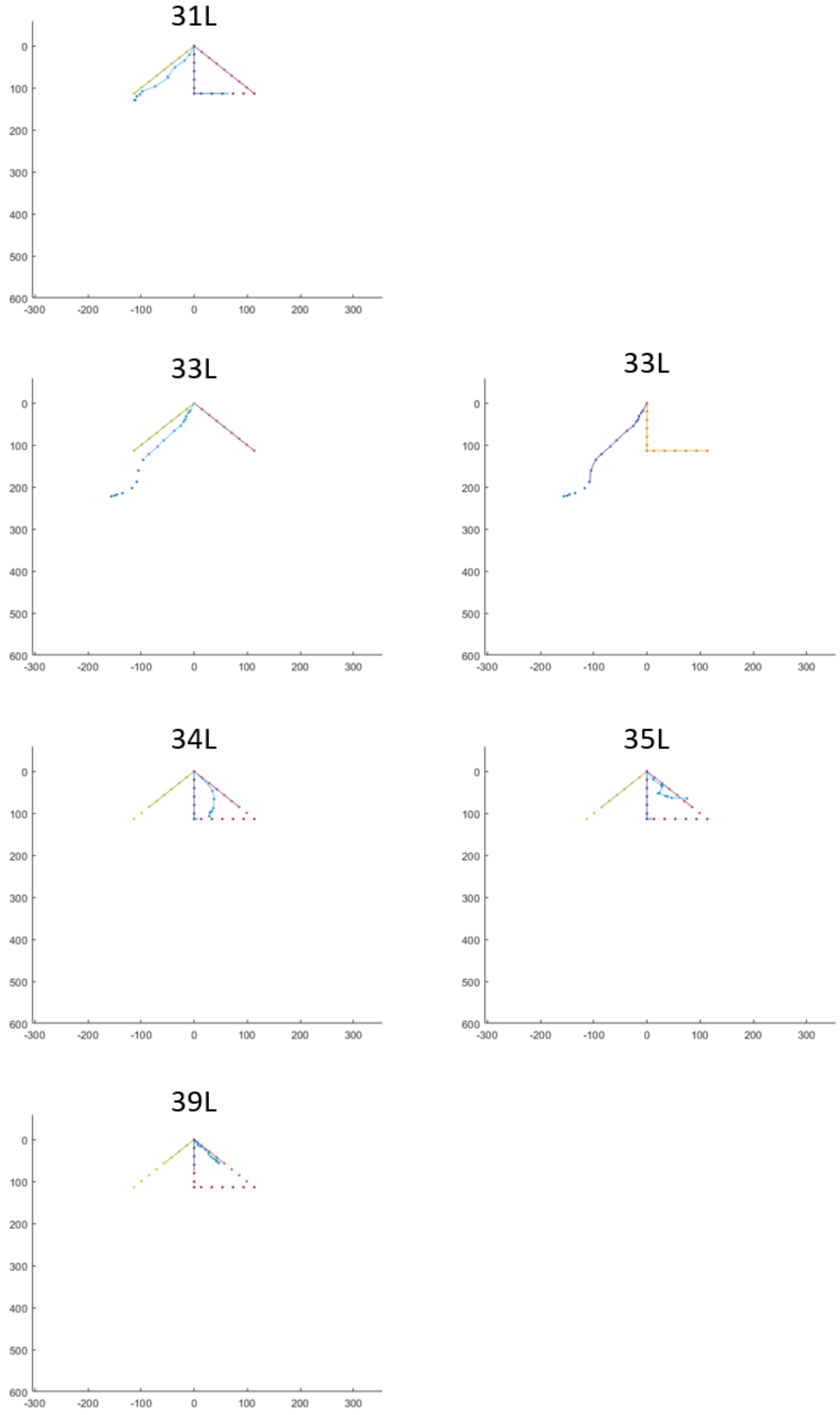

Supplementary Figure S2a: Fish and model trajectories interpolation. The sinuous path represents the fish trajectory after lateral displacement, the straight lines going down right represent the PI trajectory model, the straight lines going down left represent the APC trajectory model, the L shaped path represent the RR trajectory. Dots represent the fish position (coordinates) taken every second or the model coordinate. Lines represent the cropped trajectories implemented with 1000 points.

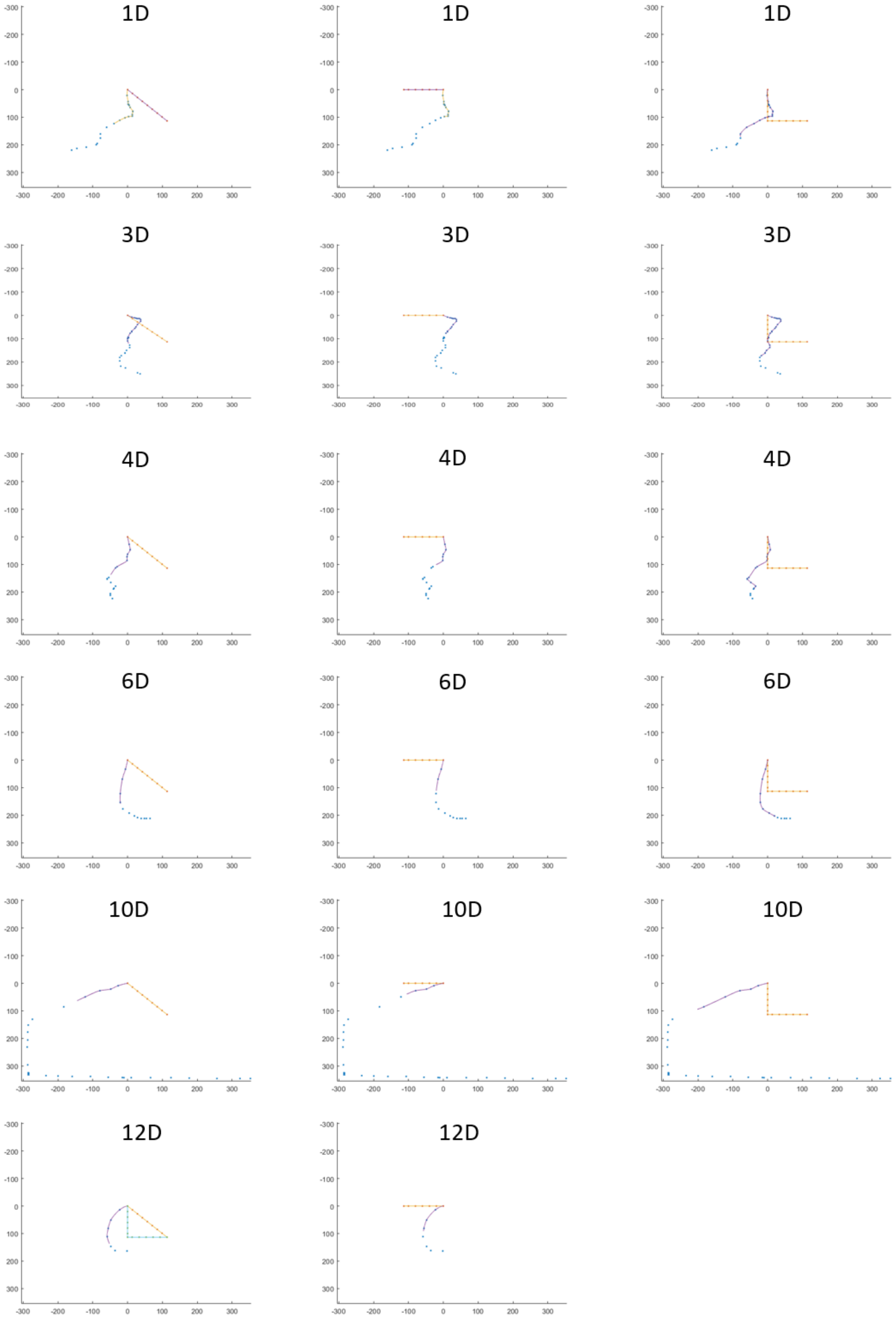
1D
1D
1D
3D
3D
3D
4D
4D
4D
6D
6D
6D
10D
10D
10D
12D
12D

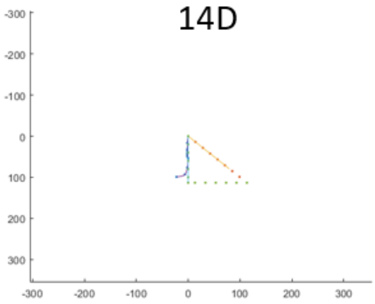
14D
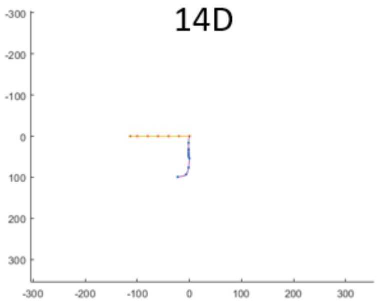
14D
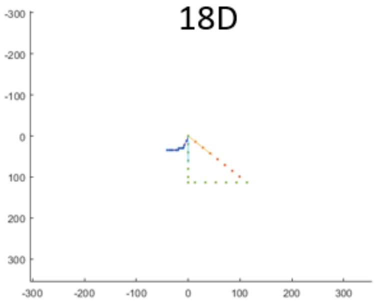
18D
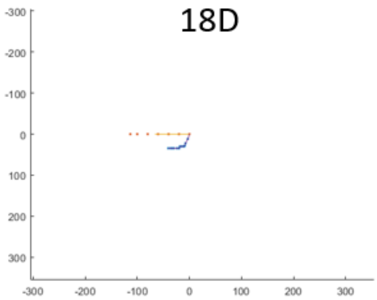
18D
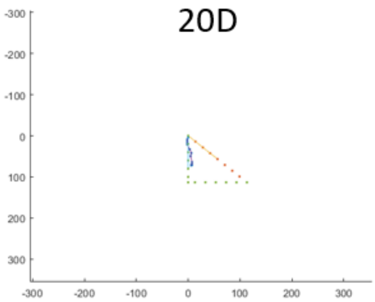
20D
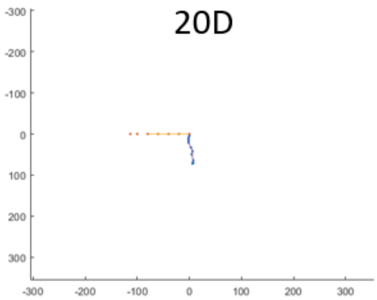
20D
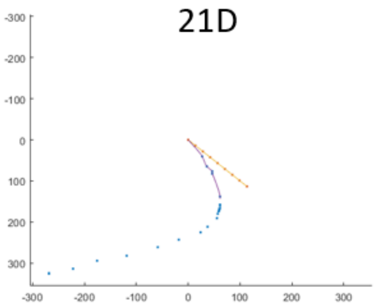
21D
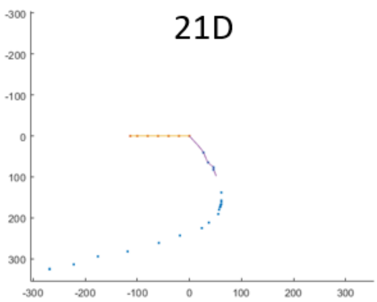
21D
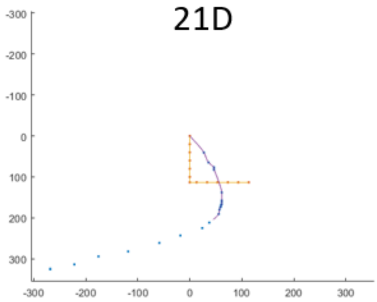
21D
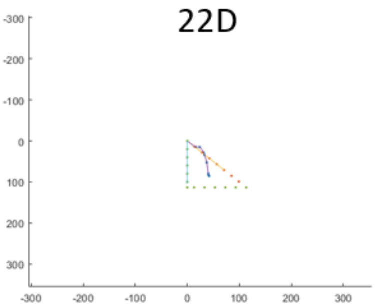
22D
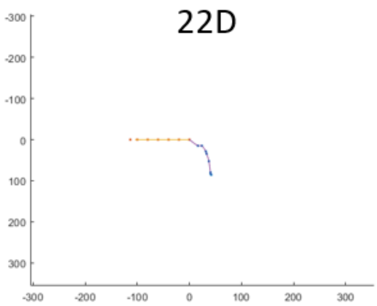
22D
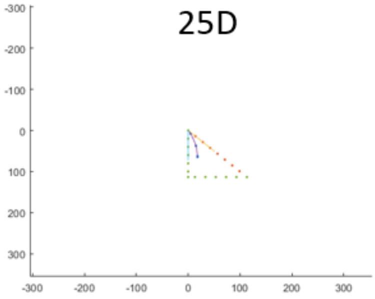
25D
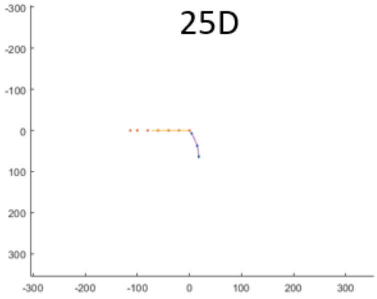
25D

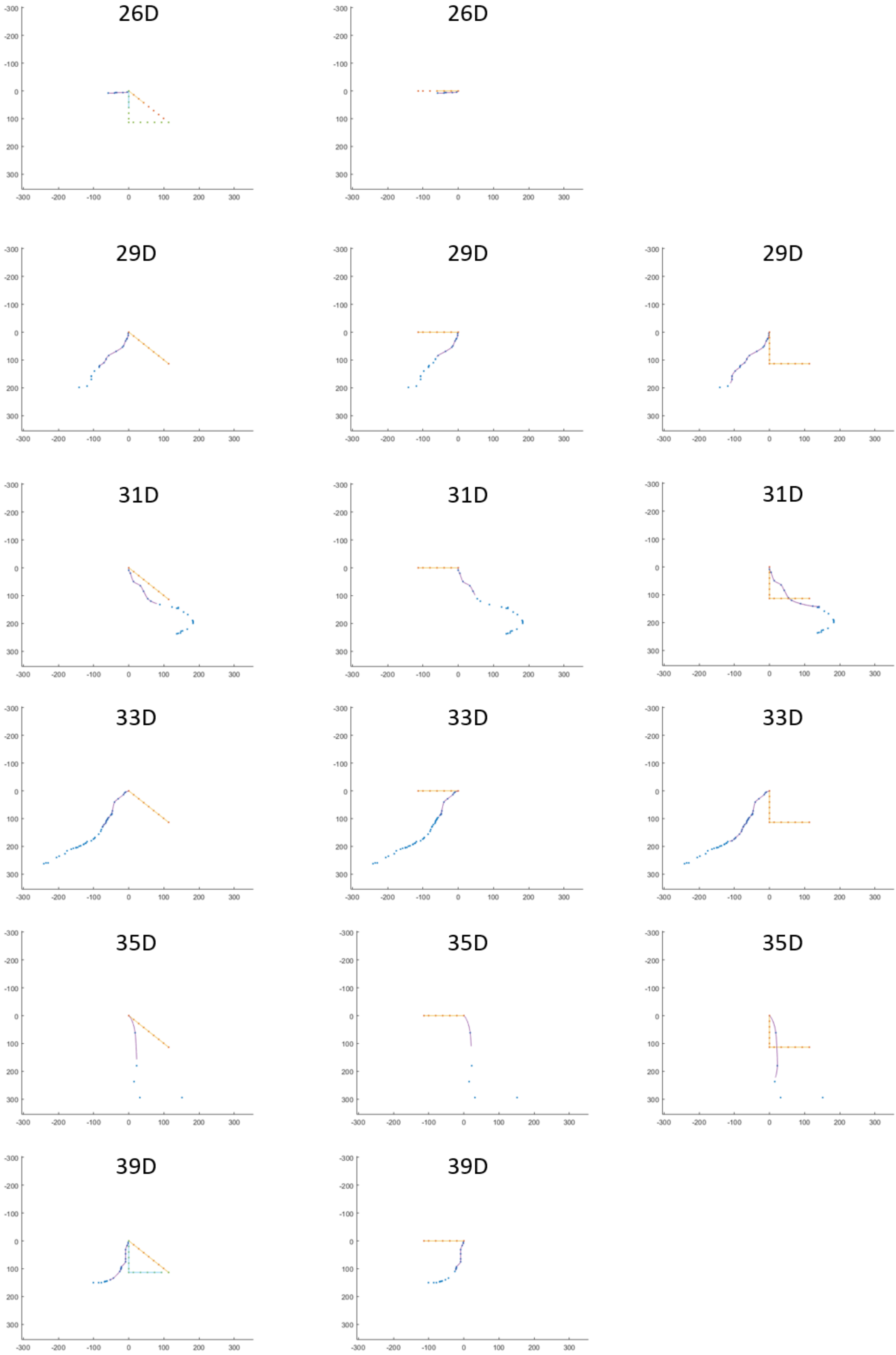

26D
26D
29D
29D
29D
31D
31D
31D
33D
33D
33D
35D
35D
35D
39D
39D
-300 -200 -100 0 100 200 300

Supplementary Figure S2b: Fish and model trajectories interpolation. The sinuous path represents the fish trajectory after diagonal displacement, the straight lines going down right represent the PI trajectory model, the straight lines going down left represent the AC trajectory model, the L shaped paths represent the RR trajectory. Dots represent the fish position (coordinates) taken every second or the model coordinate. Lines represent the cropped trajectories implemented with 1000 points.

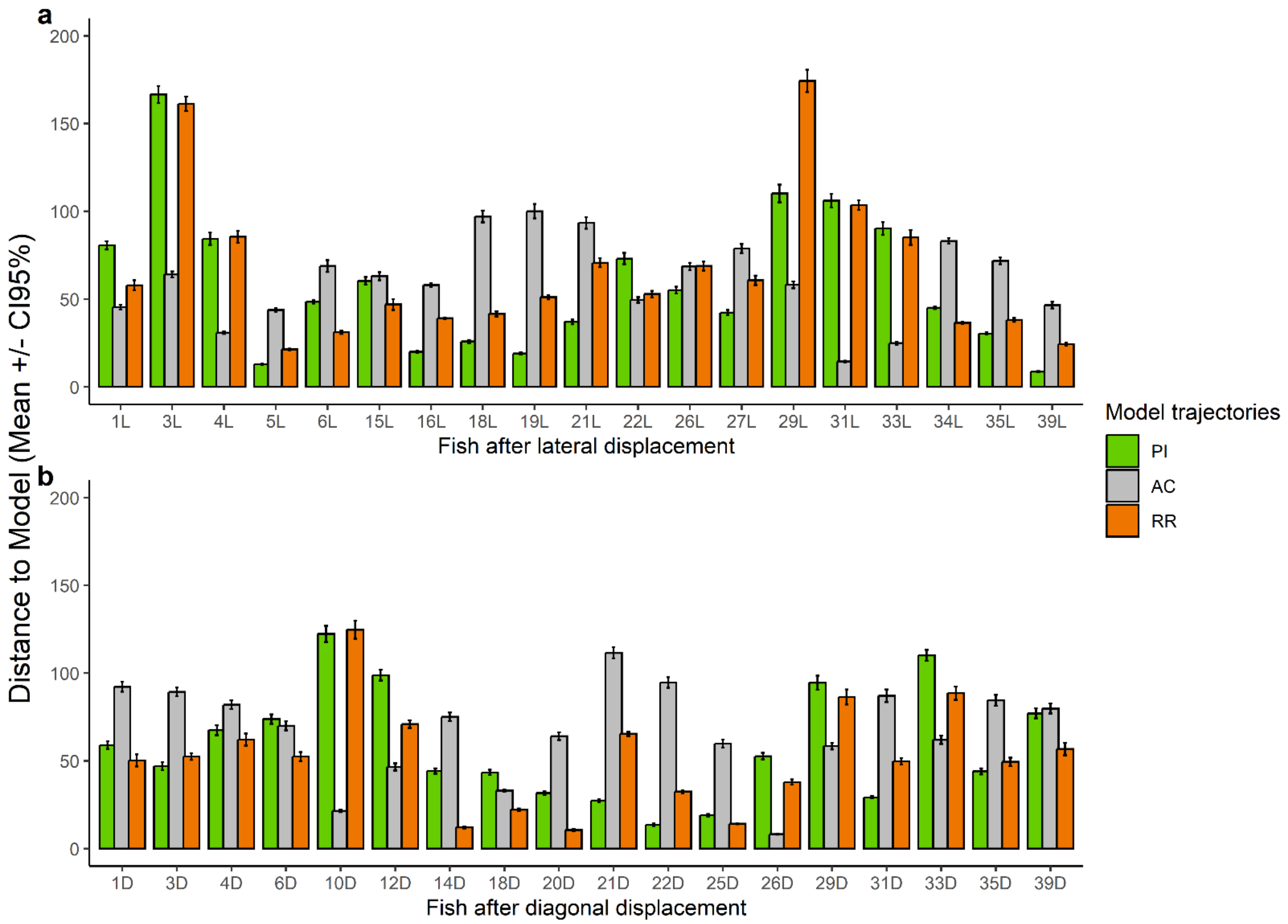

Supplementary Figure S3: Average distance ± $CI_{95\%}$ between the fish trajectories and the model trajectories after a) lateral displacement or b) diagonal displacement. The average distances were calculated over the 10000 interpolated data points from the fish trajectory and each of the model trajectories. PI= Path integration, AC= Allothetic place cues, RR=Route recapitulation.

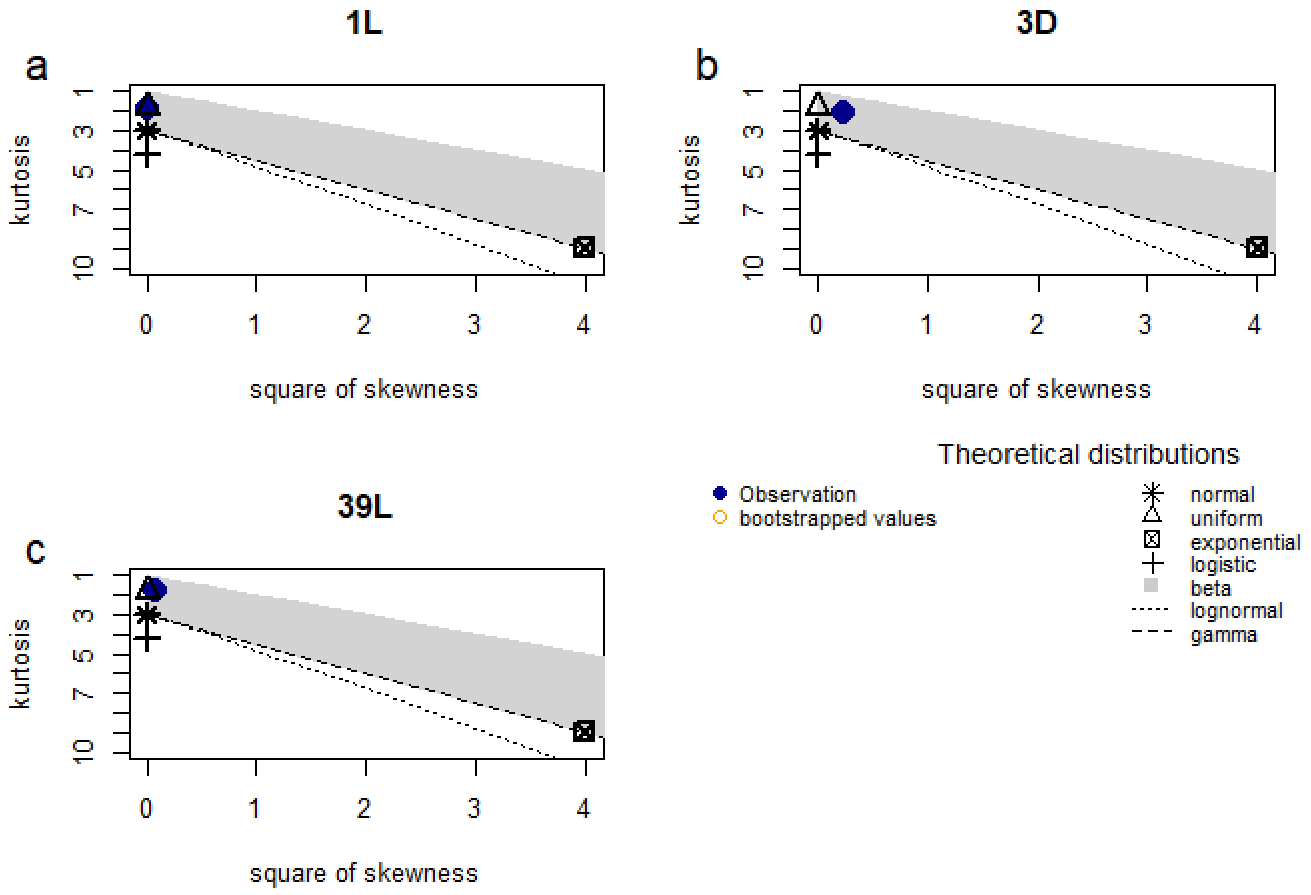

Supplementary Figure S4: Cullen and Frey figures showing the distribution of the distance between the fish 1L (S4a), 3D (S4b) and 39L (S4c) trajectories and the 10000 randomly generated trajectories.

Supplementary References

Fitak, R. R., & Johnsen, S. (2017). Bringing the analysis of animal orientation data full circle: Model-based approaches with maximum likelihood. *Journal of Experimental Biology*, *220*(21), 3878–3882. https://doi.org/10.1242/jeb.167056
