# Supplementary material for: Taking a shortcut: what mechanisms do fish use?
Source: Commun Biol. 2024 May 16;7:578. doi: 10.1038/s42003-024-06179-5 (PMC11099040; doi:10.1038/s42003-024-06179-5)
Supplement: Supplementary file 3 — Reporting Summary [file 42003_2024_6179_MOESM3_ESM.pdf]

nature portfolio

Corresponding author(s): Adelaide Sibeaux

Last updated by author(s): Mar 25, 2024

Reporting Summary

Nature Portfolio wishes to improve the reproducibility of the work that we publish. This form provides structure for consistency and transparency in reporting. For further information on Nature Portfolio policies, see our Editorial Policies and the Editorial Policy Checklist.

Statistics

For all statistical analyses, confirm that the following items are present in the figure legend, table legend, main text, or Methods section.

n/a	Confirmed	
☐	☒	The exact sample size (*n*) for each experimental group/condition, given as a discrete number and unit of measurement
☐	☒	A statement on whether measurements were taken from distinct samples or whether the same sample was measured repeatedly
☐	☒	The statistical test(s) used AND whether they are one- or two-sided<br>*Only common tests should be described solely by name; describe more complex techniques in the Methods section.*
☐	☒	A description of all covariates tested
☐	☒	A description of any assumptions or corrections, such as tests of normality and adjustment for multiple comparisons
☐	☒	A full description of the statistical parameters including central tendency (e.g. means) or other basic estimates (e.g. regression coefficient) AND variation (e.g. standard deviation) or associated estimates of uncertainty (e.g. confidence intervals)
☐	☒	For null hypothesis testing, the test statistic (e.g. *F*, *t*, *r*) with confidence intervals, effect sizes, degrees of freedom and *P* value noted<br>*Give P values as exact values whenever suitable.*
☒	☐	For Bayesian analysis, information on the choice of priors and Markov chain Monte Carlo settings
☒	☐	For hierarchical and complex designs, identification of the appropriate level for tests and full reporting of outcomes
☒	☐	Estimates of effect sizes (e.g. Cohen's *d*, Pearson's *r*), indicating how they were calculated

Our web collection on statistics for biologists contains articles on many of the points above.

Software and code

Policy information about availability of computer code

Data collection	Matlab code used to collect data is shared through Dryad open data repository (DOI: 10.5061/dryad.83bk3j9xr _ private for per review).
Data analysis	Matlab and R code used for the analyses and for the figures is shared through Dryad open data repository (DOI: 10.5061/dryad.83bk3j9xr _ private for per review).

For manuscripts utilizing custom algorithms or software that are central to the research but not yet described in published literature, software must be made available to editors and reviewers. We strongly encourage code deposition in a community repository (e.g. GitHub). See the Nature Portfolio guidelines for submitting code & software for further information.

Data

Policy information about availability of data

All manuscripts must include a data availability statement. This statement should provide the following information, where applicable:

- Accession codes, unique identifiers, or web links for publicly available datasets
- A description of any restrictions on data availability
- For clinical datasets or third party data, please ensure that the statement adheres to our policy

All data used in the analysis is shared through Dryad open data repository (DOI: 10.5061/dryad.83bk3j9xr _ private for per review).

Human research participants

Policy information about studies involving human research participants and Sex and Gender in Research.

Reporting on sex and gender	NA
Population characteristics	NA
Recruitment	NA
Ethics oversight	NA

Note that full information on the approval of the study protocol must also be provided in the manuscript.

Field-specific reporting

Please select the one below that is the best fit for your research. If you are not sure, read the appropriate sections before making your selection.

☐ Life sciences ☒ Behavioural & social sciences ☐ Ecological, evolutionary & environmental sciences

For a reference copy of the document with all sections, see nature.com/documents/nr-reporting-summary-flat.pdf

Behavioural & social sciences study design

All studies must disclose on these points even when the disclosure is negative.

Study description	We developed a behavioural paradigm to test navigational strategies availables to cichlids (Lamprologus ocellatus) following a displacement test. After the fish were attracted and trap into a reward chamber using a food reward they were displaced, released and free movement were observed for 5minutes. Each fish was tested twice. The analyses included movement tracking, frequentist statistics and circular analysis.
Research sample	We tested 40 fish to be able to discriminate between the different navigational strategies used. These fish show a low engegement rate with only 24 fish entering and exiting the chamber. The relatively low engagement rate highlights the attachment of male L. ocellatus to their shells, which was a prerequisite for testing path integration.
Sampling strategy	We extracted all possible data from our 24 participant fish.
Data collection	All trials were recorded using an overhead web camera (ELP Webcam 10-megapixel, Model X000VD0KT5). Videos were manually processed using custom code written for MATLAB version R2022a, MathWorks Inc. Every second, the position of the fish was identified by clicking on the head of the fish. X and Y coordinates were then extracted and used to produce a continuous 2D trajectory. Fish movements were analysed for one minute after exiting the chamber.
Timing	All experiment were performed between the 30th November 2020 and the 4th April 2021. Only one fish was tested per day. The fish had a break of two weeks before being tested in another trial.
Data exclusions	Once the fish exited the reward chamber, when their distance travelled was shorter than the chamber diameter the trial was discarded (n=10 trials out of 80 trials performed).
Non-participation	Only 24 fish out of 40 entering and exiting the chamber and allowed us to collect data. The relatively low engagement rate highlights the attachment of male L. ocellatus to their shells, which was a prerequisite for testing path integration.
Randomization	All fish were tested in the same condition. Grouping based on their navigational strategies was performed a posteriori

Reporting for specific materials, systems and methods

We require information from authors about some types of materials, experimental systems and methods used in many studies. Here, indicate whether each material, system or method listed is relevant to your study. If you are not sure if a list item applies to your research, read the appropriate section before selecting a response.

Materials & experimental systems

n/a	Involved in the study
☒	☐ Antibodies
☒	☐ Eukaryotic cell lines
☒	☐ Palaeontology and archaeology
☐	☒ Animals and other organisms
☒	☐ Clinical data
☒	☐ Dual use research of concern

Methods

n/a	Involved in the study
☒	☐ ChIP-seq
☒	☐ Flow cytometry
☒	☐ MRI-based neuroimaging

Animals and other research organisms

Policy information about studies involving animals; ARRIVE guidelines recommended for reporting animal research, and Sex and Gender in Research

Laboratory animals	Lamprologus ocellatus, were sourced from a captive-bred laboratory population (individual age are available on the data file _dryad depository)
Wild animals	NA
Reporting on sex	We used males individuals for this study as their attachment to their home shell was a requirement for the study. (indicated lines 309)
Field-collected samples	NA
Ethics oversight	The study was approved by the University of Oxford's Animal Welfare and Ethical Review Body (AWERB) (project code: APA/1/5/ZOO/NASPA/Burt/DistanceEstimation).

Note that full information on the approval of the study protocol must also be provided in the manuscript.
